# Supplementary material for: Spatially aware clustering of ion images in mass spectrometry imaging data using deep learning
Source: Anal Bioanal Chem. 2021 Mar 1;413(10):2803–19. doi: 10.1007/s00216-021-03179-w (PMC8007517; doi:10.1007/s00216-021-03179-w)
Supplement: Supplementary file 1 — (PDF 3.06 MB) [file 216_2021_3179_MOESM1_ESM.pdf]

Supplementary Information

I. Relative isotope ratio (RIR) metric examples:

We conducted some experiments on both data sets to show that the RIR metric can reasonably determine whether two ion images are an isotope by assessing their spectral and spatial expression. In the following examples using the lymph node and mouse kidney data, the RIR metric detects 3 and 2 ion images respectively as an isotope. Besides, we also show some example ion images from the same cluster, but they are not detected as isotope by the RIR metric.

i. Lymph node data:

a. Detect as isotope:

(760.6285939640838, 761.6440294571262),
(761.6440294571262, 762.6594649501685),

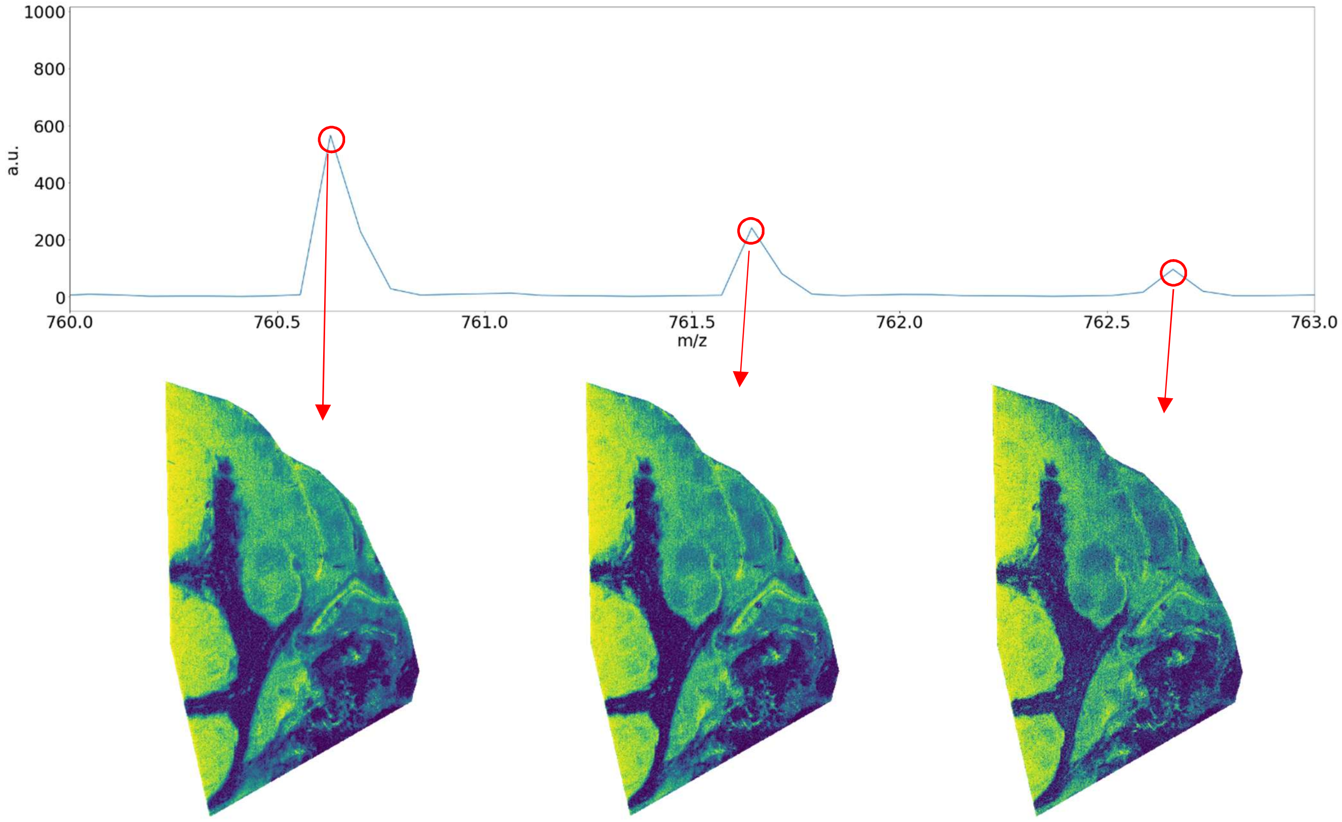

760.63 761.64 762.66

b. Example ion images (belongs to the same cluster but not the isotope):

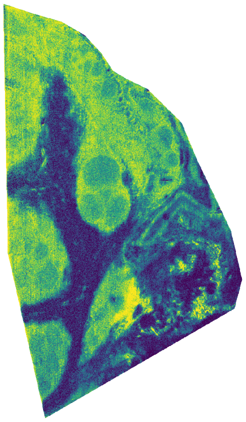

m/z: 725.60

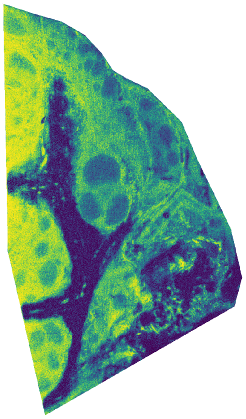

m/z: 703.62

ii. Mouse kidney data:

a. Detect as isotope: (856.5643, 857.56824)

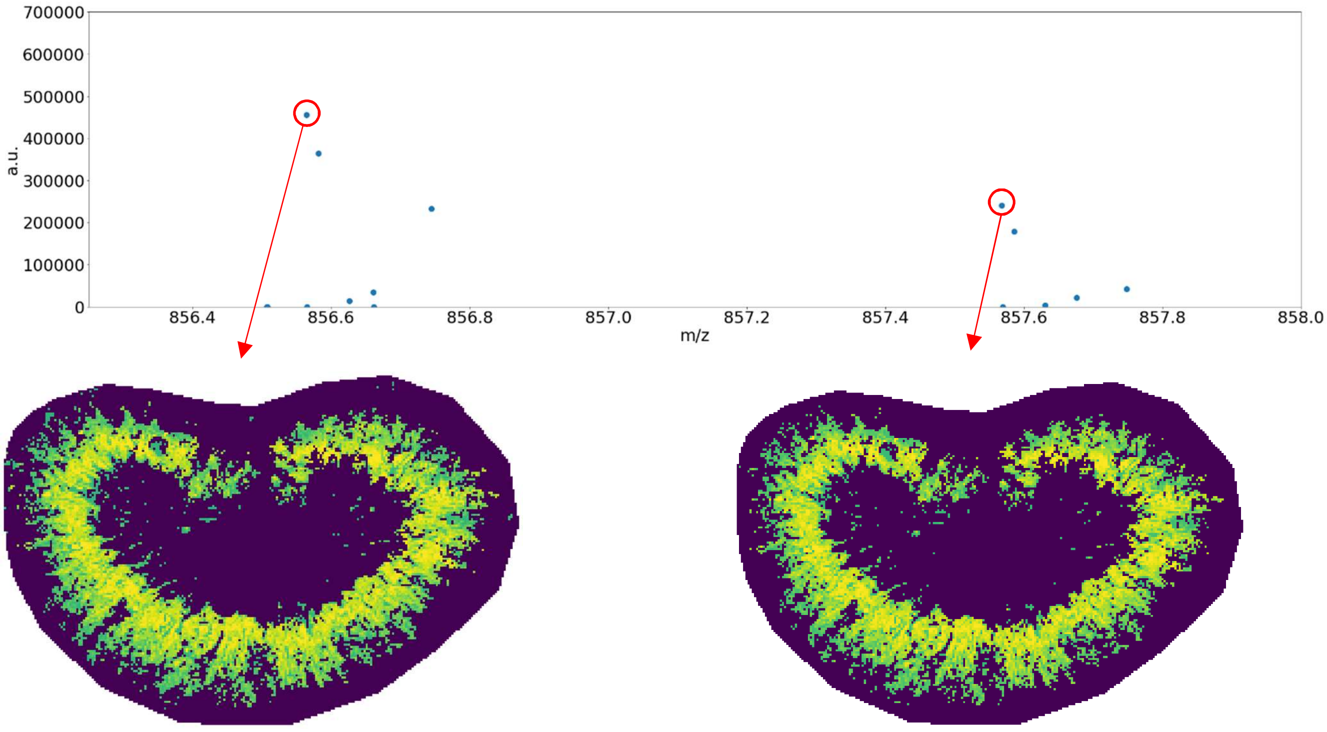

856.5643

857.56824

b. Example ion image (belongs to the same cluster but not the isotope):

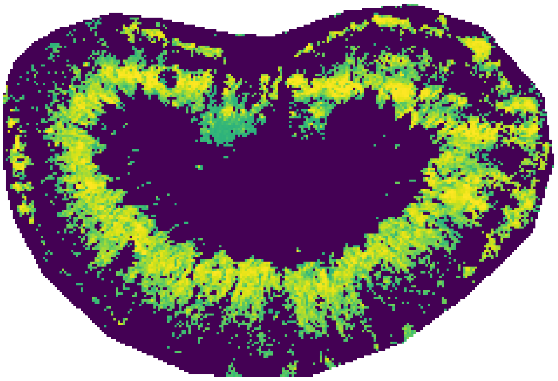

m/z: 819.61

II. Used data sets from ColocML:

Those are the dataset_Id of the data sets we used. Only those sets contain the spatial information and experts golden standards:

```
2017-01-26_09h08m49s
2017-07-18_17h21m08s (3 pairs of sets)
2018-09-14_22h35m17s
2018-11-28_14h47m01s
2018-12-04_14h23m49s
2018-12-12_12h51m09s
2018-12-18_12h33m17s
2019-01-01_01h09m28s
2019-01-04_00h50m49s
2019-01-04_01h25m24s
2019-01-04_20h32m07s
2019-01-05_02h22m18s
2019-01-17_19h54m30s (2 pairs of sets)
2019-01-21_22h44m45s
2019-01-22_03h04m17s
2019-01-23_00h34m56s
2019-01-25_11h22m48s
2019-01-25_11h24m31s (2 pairs of sets)
2019-01-25_11h26m55s
2019-03-12_08h57m03s
2019-03-12_10h14m51s
2019-03-19_17h21m15s (2 pairs of sets)
2019-03-22_10h16m54s
```

III. Examples from ColocML data:

i. datasetId = ‘2017-01-26_09h08m49s’

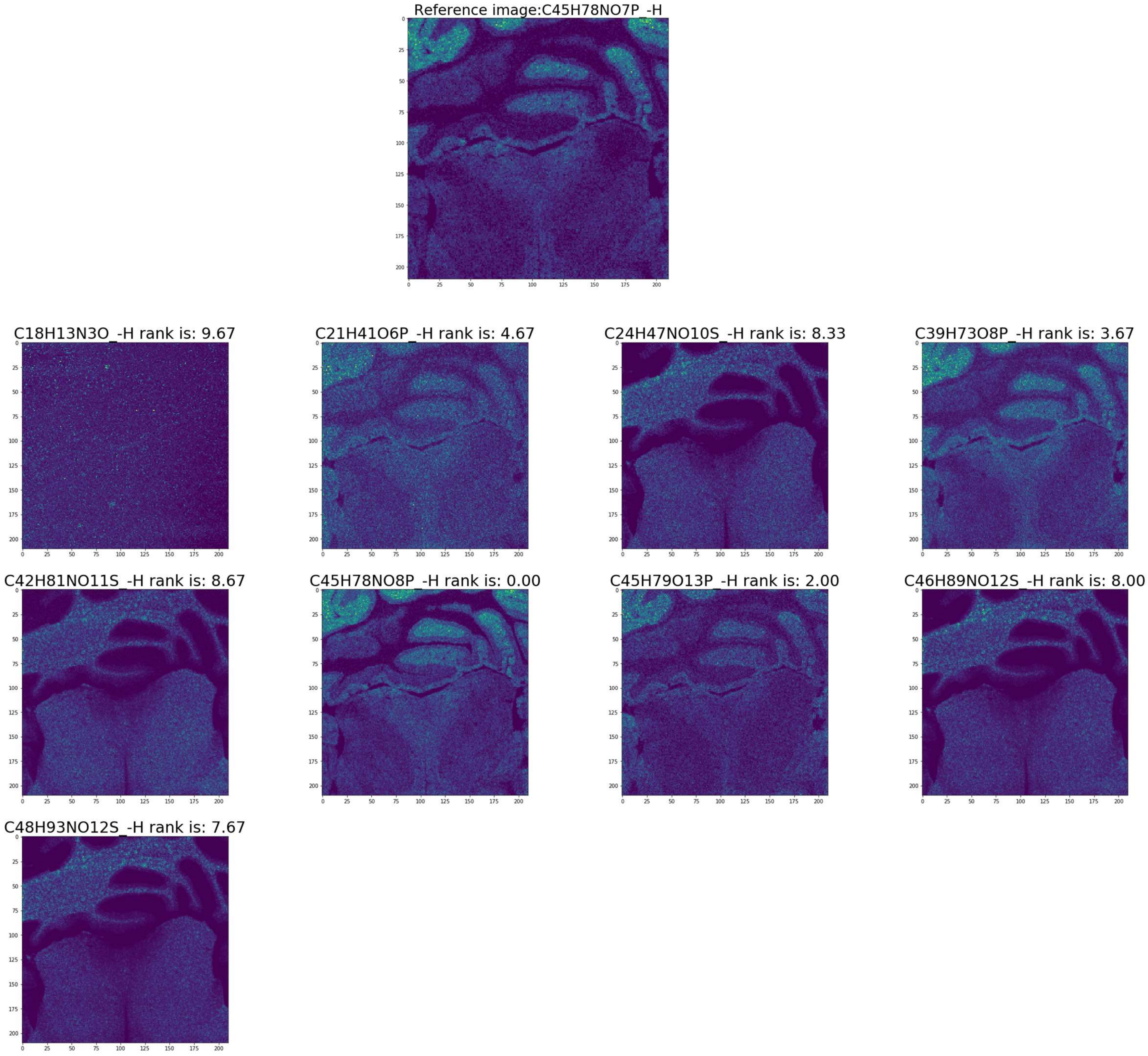

ii. datasetId = ‘2017-07-18_17h21m08s’

a. Set 1:

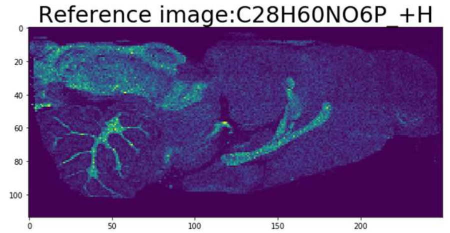

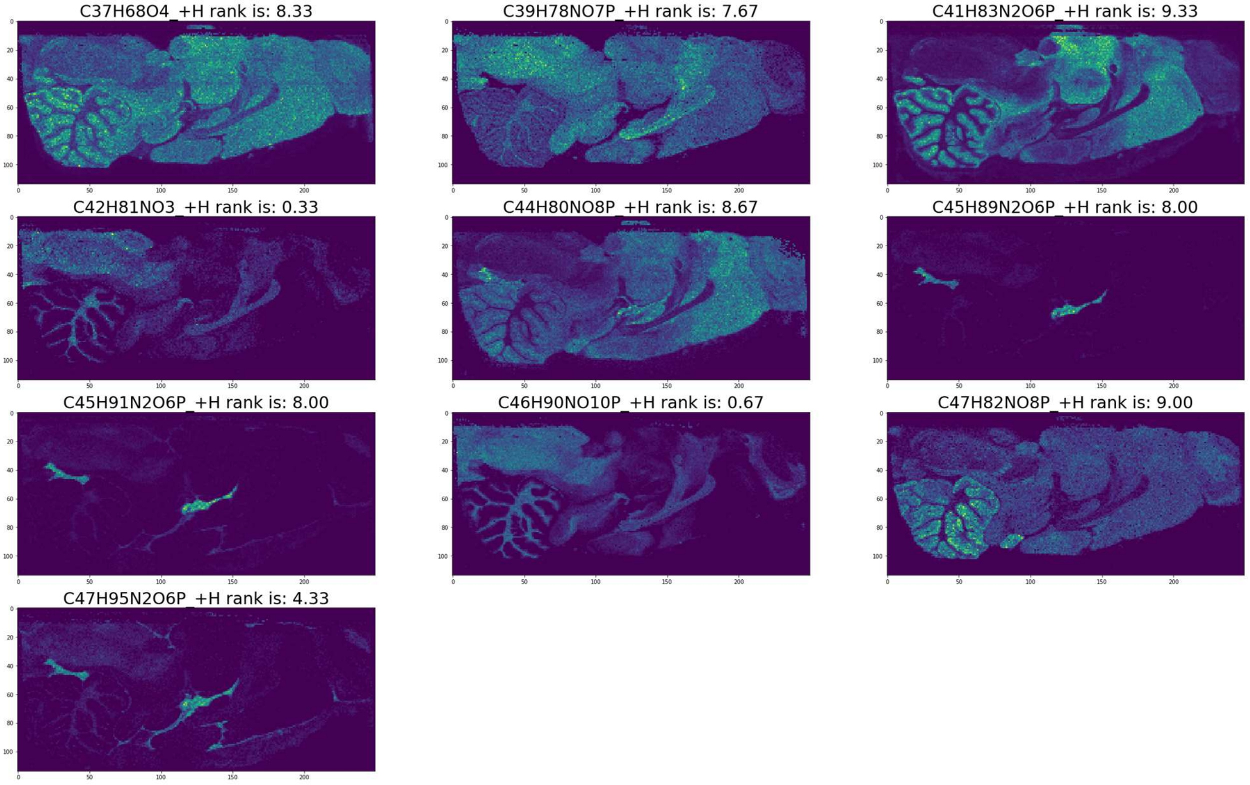

b. Set 2:

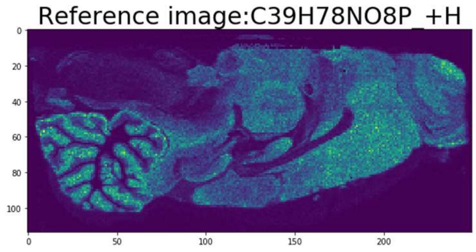

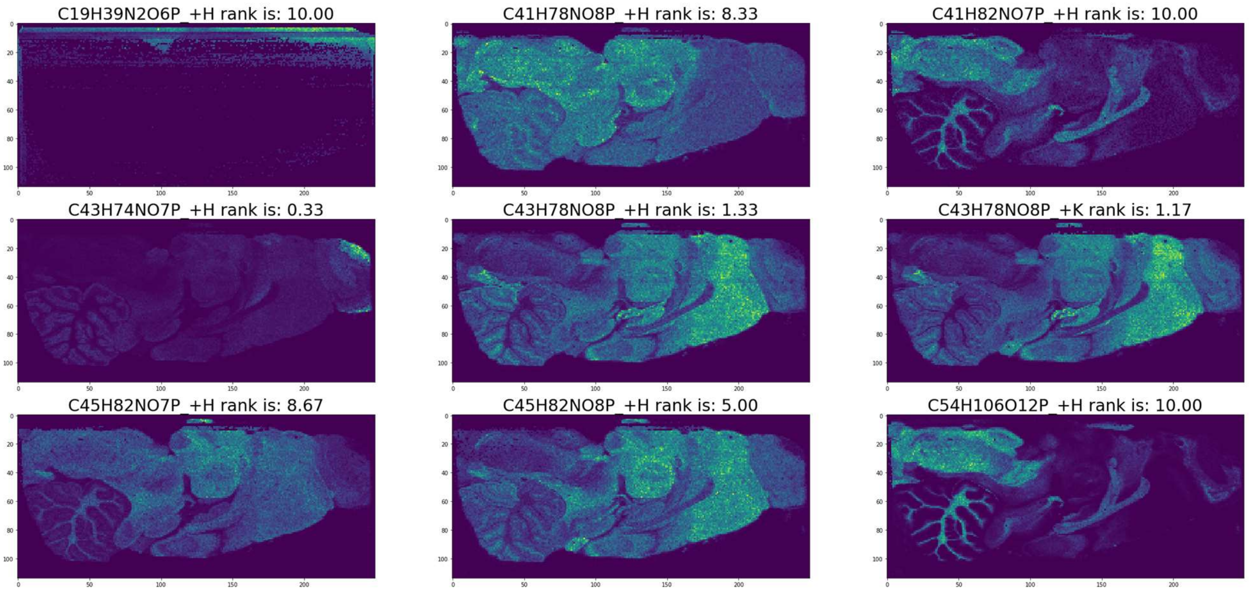
C19H39N2O6P_+H rank is: 10.00
C41H78NO8P_+H rank is: 8.33
C41H82NO7P_+H rank is: 10.00
C43H74NO7P_+H rank is: 0.33
C43H78NO8P_+H rank is: 1.33
C43H78NO8P_+K rank is: 1.17
C45H82NO7P_+H rank is: 8.67
C45H82NO8P_+H rank is: 5.00
C54H106O12P_+H rank is: 10.00

c. Set 3:

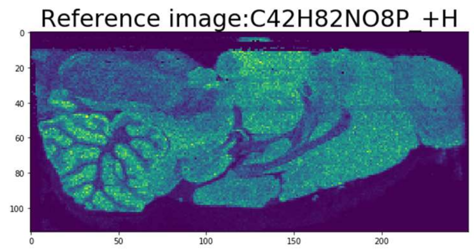
Reference image:C42H82NO8P_+H

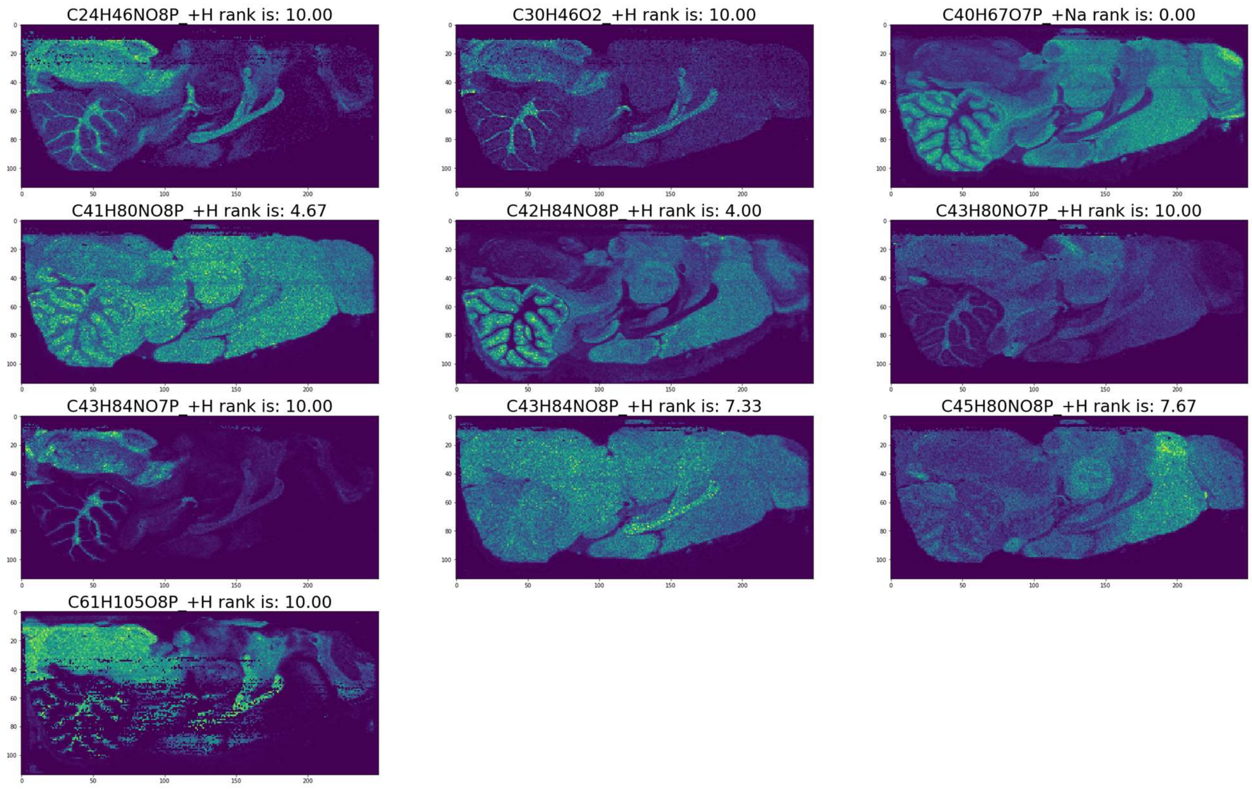
C24H46NO8P_+H rank is: 10.00
C30H46O2_+H rank is: 10.00
C40H67O7P_+Na rank is: 0.00
C41H80NO8P_+H rank is: 4.67
C42H84NO8P_+H rank is: 4.00
C43H80NO7P_+H rank is: 10.00
C43H84NO7P_+H rank is: 10.00
C43H84NO8P_+H rank is: 7.33
C45H80NO8P_+H rank is: 7.67
C61H105O8P_+H rank is: 10.00
